# Correlation between Echocardiography and Cardiac Catheterization for the Assessment of Pulmonary Hypertension in Pediatric Patients

**DOI:** 10.7759/cureus.5511

**Published:** 2019-08-29

**Authors:** Arshad Sohail, Hussain B Korejo, Abdul Sattar Shaikh, Aliya Ahsan, Ram Chand, Najma Patel, Musa Karim

**Affiliations:** 1 Pediatric Cardiology, National Institute of Cardiovascular Diseases, Karachi, PAK; 2 Cardiology, National Institute of Cardiovascular Disease, Karachi, PAK; 3 Paediatric Cardiology, National Institute of Cardiovascular Diseases, Karachi, PAK; 4 Miscellaneous, National Institute of Cardiovascular Diseases, Karachi, PAK

**Keywords:** pulmonary hypertension, echocardiography, cardiac catheterization, pulmonary artery pressure, correlation

## Abstract

Introduction

Cardiac catheterization is widely considered the “gold standard” for the diagnosis of pulmonary hypertension. However, its routine use is limited due to its invasive nature. Therefore, the aim of this study was to evaluate the correlation between pulmonary artery pressures obtained by various parameters of transthoracic echocardiography and cardiac catheterization.

Methods

This study includes 50 consecutive patients with intracardiac shunt lesions diagnosed with severe pulmonary hypertension on echocardiography and admitted for cardiac catheterization at the National Institute of Cardiovascular Diseases (NICVD) in Karachi, Pakistan. Cardiac catheterization and transthoracic echocardiography were performed in all patients simultaneously and systolic (sPAP) and mean pulmonary artery pressure (mPAP) were assessed with both modalities. Correlations and agreement, in terms of Bland-Altman plot, were computed between both modalities for sPAP and mPAP.

Results

Out of 50 patients, 46% (23) were male and mean age was 7.49 ± 4.45 years. On cardiac catheterization, sPAP was 93.92 ± 17.91 mmHg and mPAP was 67.0 ± 14.28 mmHg. Correlation between cardiac catheterization and echocardiography for the assessment of sPAP was 0.917 (p<0.001), and mPAP was 0.832 (p<0.001) for mean gradient of tricuspid regurgitation (PGTRmean), 0.749 (p<0.001) for peak gradient of pulmonary regurgitation (PGPRpeak), 0.691 (p<0.001) for Acceleration time across right ventricular outflow tract (RVOT), and 0.752 (p<0.001) for end gradient of pulmonary regurgitation (PGPRend). Bland-Altman plot showed moderate agreement between two modalities.

Conclusion

A positive but modest correlation was observed between hemodynamic parameters of transthoracic echocardiography and cardiac catheterization for assessment of pulmonary artery pressures. Transthoracic echocardiography can reliably be used as an initial non-invasive modality for the assessment of pulmonary artery hypertension and can obviate the need of right heart catheterization in some patient especially with mild pulmonary hypertension.

## Introduction

Pulmonary hypertension (PH), according to the European Society of Cardiology (ESC) guidelines for diagnosis and treatment of pulmonary hypertension, is defined as mean pulmonary artery pressure (mPAP) ≥ 25 mmHg at rest assessed by right heart catheterization as gold standard investigation [1,2]. Pulmonary artery hypertension occurs frequently in congenital heart disease with intra or extracardiac left to right shunt [3].

Cardiac catheterization is widely considered “gold standard” for the diagnosis of pulmonary hypertension [4]. However, its routine use is limited due to its invasive nature, inherent complications and incurring cost [2,5,6]. Therefore it cannot be performed in every patient without clear indication. Compared with a right heart catheterization, transthoracic echocardiography is non-invasive, inexpensive and widely available, and is therefore attractive not only as an initial screening tool for pulmonary hypertension assessment but also as a method of monitoring disease progression over time [4,7-9].

Different studies have compared different parameters of echocardiography for pulmonary artery pressure assessment in pulmonary hypertension patients with cardiac catheterization and found that echocardiographic assessment of peak and mean pulmonary artery pressure (mPAP) is closely related to that assessed by cardiac catheterization [10]. As PH is defined by mean pulmonary artery pressure and it is directly related to pulmonary vascular resistance, therefore it is of utmost important to take it on a routine basis in patients with shunt lesions to assess operability and may obviate the need for cardiac catheterization in some patients. However, it is not routinely taken, especially by the mean gradient of tricuspid regurgitation (PGTRmean) and acceleration time across right ventricular outflow tract (RVOT). Thus, this study was designed to highlight the importance of above parameters in routine echocardiography. The aim of this study was to evaluate the correlation between pulmonary artery pressures obtained by various parameters of transthoracic echocardiography (TTE) and cardiac catheterization.

## Materials and methods

In this cross-sectional study, 50 children, aged 1.5 years to 15 years presented at pediatric cardiology department of National Institute of Cardiovascular Diseases Karachi, Pakistan (NICVD) during the study period of January 2018 to July 2018. Patients were selected by using the non-probability consecutive sampling technique. Patients diagnosed with severe pulmonary artery hypertension (PAH) associated with shunt lesion, such as ventricular septal defect (VSD), VSD with atrial septal defects (ASD)/ patent ductus arteriosus (PDA), PDA and PDA with ASD on echocardiography and scheduled for right heart catheterization were included in this study. Consent for the participation and confidentiality was obtained from the parents/guardian of the patients. Patients with cyanotic heart disease, ventricular dysfunction, dysrhythmia, free tricuspid regurgitation (TR), and associated valvular and other cardiac anomalies were excluded from this study. Cardiac catheterization and echocardiography were performed in all patients simultaneously and echocardiography findings such as; peak gradient of tricuspid regurgitation (PGTRpeak), mean gradient of tricuspid regurgitation (PGTRmean), peak gradient of pulmonary regurgitation (PGPRpeak), end gradient of pulmonary regurgitation (PGPRend), right atrial pressure (RAP), and acceleration time across right ventricular outflow tract (RVOT) and right heart catheterization (RHC) finding such as; systolic (sPAP), mean pulmonary artery pressure (mPAP), and mean right atrial pressure (RAP) were recorded on a predefined structural proforma.

Echocardiographic examinations were performed by Toshiba Xario 200 (Toshiba America Medical Systems, Inc.) ultrasound system with five and six MHZ phased array transducer within a half-hour of cardiac catheterization. Echocardiography was performed by a single experienced sonographer, totally blind to the cardiac catheterization data of the patient. Sweep speed of 100 was kept for the Doppler and an average of three findings were calculated. Assessment of pulmonary artery pressures i.e. systolic and mean, was done by using different parameters including tricuspid valve regurgitation velocity, pulmonary valve regurgitation velocity and acceleration time across right ventricular outflow tract [6]. The machine automatically converts the velocity to pressure gradient by simplified Bernoulli equation, ∆P = 4Vmax2, and right atrial pressure (RAP) was added, which was calculated from inferior vena cava (IVC) size and collapsability [8,11,12]. This data was finally analyzed by one expert cardiologist.

For Systolic pulmonary artery pressure (sPAP), the tricuspid valve was visualized properly on 2D images in parasternal short axis and the apical four-chamber view and TR jet was obtained by color Doppler. Then, by using continuous-wave Doppler, a satisfactory envelope was obtained. Peak velocity was obtained by tracing the envelope, which was automatically converted to gradient by machine as discussed above. Systolic pulmonary artery pressure was calculated by adding the right atrial pressure to peak gradient [13].

Mean Pulmonary Artery Pressure from tricuspid regurgitation (TR) was calculated by adding right atrial pressure to the mean pressure gradient across the tricuspid valve obtained by tracing TR jet as discussed above. [13]

For the calculation of mean pulmonary artery pressure by pulmonary regurgitation (PR), pulmonic valve was visualized properly in the parasternal short axis on 2D images and PR jet was obtained on color Doppler. Then, by using continuous-wave doppler, pulmonary regurgitation wave was obtained, and PRpeak and PRend velocities were measured. These were then converted to gradients as discussed above. Pulmonary artery end-diastolic pressure (PAEDP) was calculated using PAEDP= PGPRend + RAP. The mPAP was calculated from PGPRpeak and PGPRend as mPAP= PGPRpeak + RAP and mPAP = 2PAEDP/3 + systolic PAP/3 respectively [8,13].

For the calculation of Mean pulmonary artery pressure from RVOT Acceleration Time, RVOT was visualized in the parasternal short axis. Using pulse wave Doppler just proximal to the pulmonic valve, the pulse wave was obtained and acceleration time was measured by taking measurements from the peak to start of the wave. The mean pulmonary artery pressure was then calculated by using mPAP = 90 - (0.62 × ATRVOT) [13].

Cardiac catheterization was performed by an expert cardiologist under conscious sedation on the Toshiba Infinix 800v CFI (Toshiba America Medical Systems, Inc.) machine. Femoral arterial and venous catheterization were done. A Berman Angiographic Catheter or NIH Catheter was used for pressures, and Oxygen saturation of right heart chambers and Pigtail Catheter was used for left-sided chamber pressures and oxygen saturation. Pressures were obtained after proper calibration and zeroing the transducer at mid-chest level.

The sample size for the study was calculated using the reported correlation of 0.87 between systolic pulmonary artery pressure (sPAP) assessed by cardiac catheterization and echocardiography [8]. With a 95% confidence interval, expected correlation of 0.87 and a confidence interval width of 0.15, the sample size for the study was calculated as n = 46. The data was analyzed using IBM SPSS Statistics for Windows, Version 21.0. (IBM Corp., Armonk, NY, US). Summary statistics such as mean ± standard deviation (SD) and frequency (percentages) were calculated appropriately. Categorical variables such as gender, age groups, and baseline diagnosis were expressed as frequency (percentages). Continuous variables such as age, sPAP, mPAP, and mRAP were expressed as mean ± SD. The pearson correlation coefficient was calculated between the hemodynamic parameters (systolic (sPAP), and mean pulmonary artery pressure (mPAP)) of PH on echocardiography and RHC. For the assessment of agreement between the two modalities, i.e. cardiac catheterization and echocardiography, a Bland-Altman plot was computed and limits of agreement were calculated.

## Results

Total of 50 patients were included in this study, out of which 46% (23) were male patients. Mean age of the patients was 7.49 ± 4.45 years (1.5 to 15 years) with 46% (23) patients up to five years of age. In the majority (96%) of the patients , the shunt lesion was VSD. Baseline characteristics of the patients are presented in Table 1.

**Table 1:** Baseline Characteristics

Total patients	N = 50
Gender	
Male	46% (23)
Female	54% (27)
Age (years)	
Mean ± SD	7.49 ± 4.45 years
Up to 5 years	46% (23)
6 to 10 years	24% (12)
11 to 15 years	30% (15)
Baseline Diagnosis	
Ventricular septal defect (VSD)	88% (44)
VSD + Atrial septal defect (ASD)	6% (3)
Patent ductus arteriosus (PDA)	4% (2)
VSD + PDA	2% (1)

The hypothesis of normality was not rejected for all three measures, namely sPAP and mPAP with Shapiro-Wilk test p-values of ≥ 0.05. On cardiac catheterization, sPAP was 93.92 ± 17.91 mmHg (55 to 140 mmHg) and mPAP was 67.0 ± 14.28 mmHg (30 to 108 mmHg). Assessment of sPAP and mPAP on echocardiography and cardiac catheterization are presented in Table 2.

**Table 2:** Assessment of Pulmonary Artery Pressure on Echocardiography and cardiac catheterization sPAP=systolic pulmonary artery pressure, mPAP=mean pulmonary artery pressure, RAP=mean right atrial pressure, TR=tricuspid regurgitation, PR=pulmonary regurgitation, RVOT=right ventricular outflow tract

Characteristics	Mean ± SD	Not Assessed (%)
Right heart catheterization (RHC)		
sPAP (mmHg)	93.92 ± 17.91	0% (0)
mPAP (mmHg)	67.0 ± 14.28	0% (0)
mRAP (mmHg)	7.8 ± 2.54	0% (0)
Transthoracic echocardiography (TTE)		
sPAP via TR	92.15 ± 17.39	6% (3)
mPAP via TRmean	64.89 ± 13.71	8% (4)
mPAP via PRpeak	65.1 ± 10.94	0% (0)
mPAP via Acceleration Time at RVOT	62.24 ± 7.67	0% (0)
mPAP via PRend	54.37 ± 11.5	14% (7)
RAP	5.3 ± 1.2	0% (0)

Correlation between sPAP assessed on cardiac catheterization and echocardiography was 0.917 (p<0.001). Similarly mPAP assessed by echocardiography showed correlation of 0.832 (p<0.001) for PGTRmean, 0.749 (p<0.001) for PGPRpeak, 0.691 (p<0.001) for Acceleration time at RVOT and 0.752 (p<0.001) for PGPRend with that assessed by cardiac catheterization

**Figure 1:**
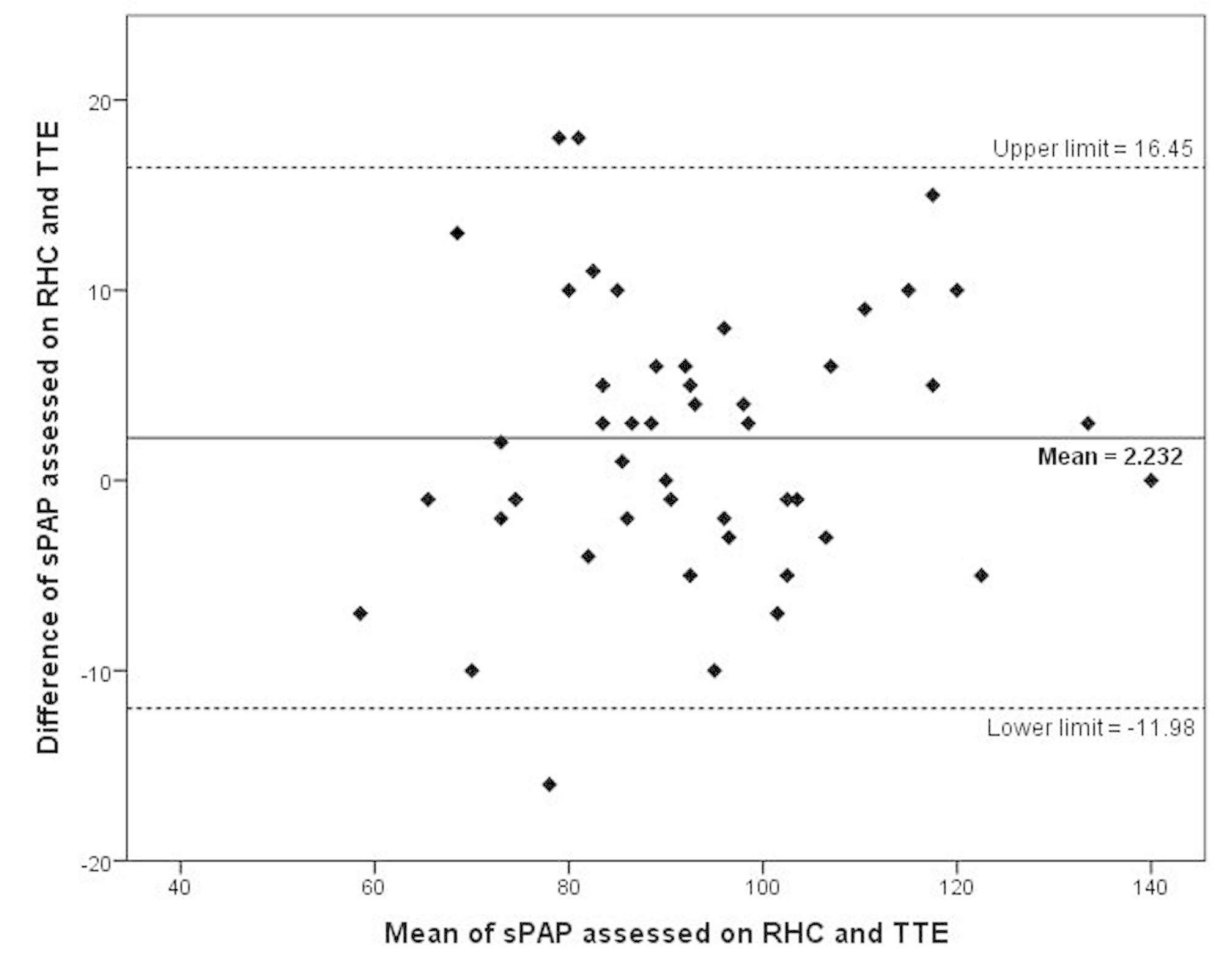
Bland-Altman plot for systolic pulmonary artery pressure (sPAP) assessed on cardiac catheterization and echocardiography TTE=transthoracic echocardiography, RHC=right heart catheterization, sPAP=systolic pulmonary artery pressure

Bland-Altman plot for sPAP assessed on cardiac catheterization and echocardiography, presented in Figure 1, showed the 95% limits of agreement was -11.98 to +16.45 mmHg, and mean difference or bias was 2.23 mmHg between the two assessments. The difference was normally distributed around the mean and limits of agreement.

**Figure 2:**
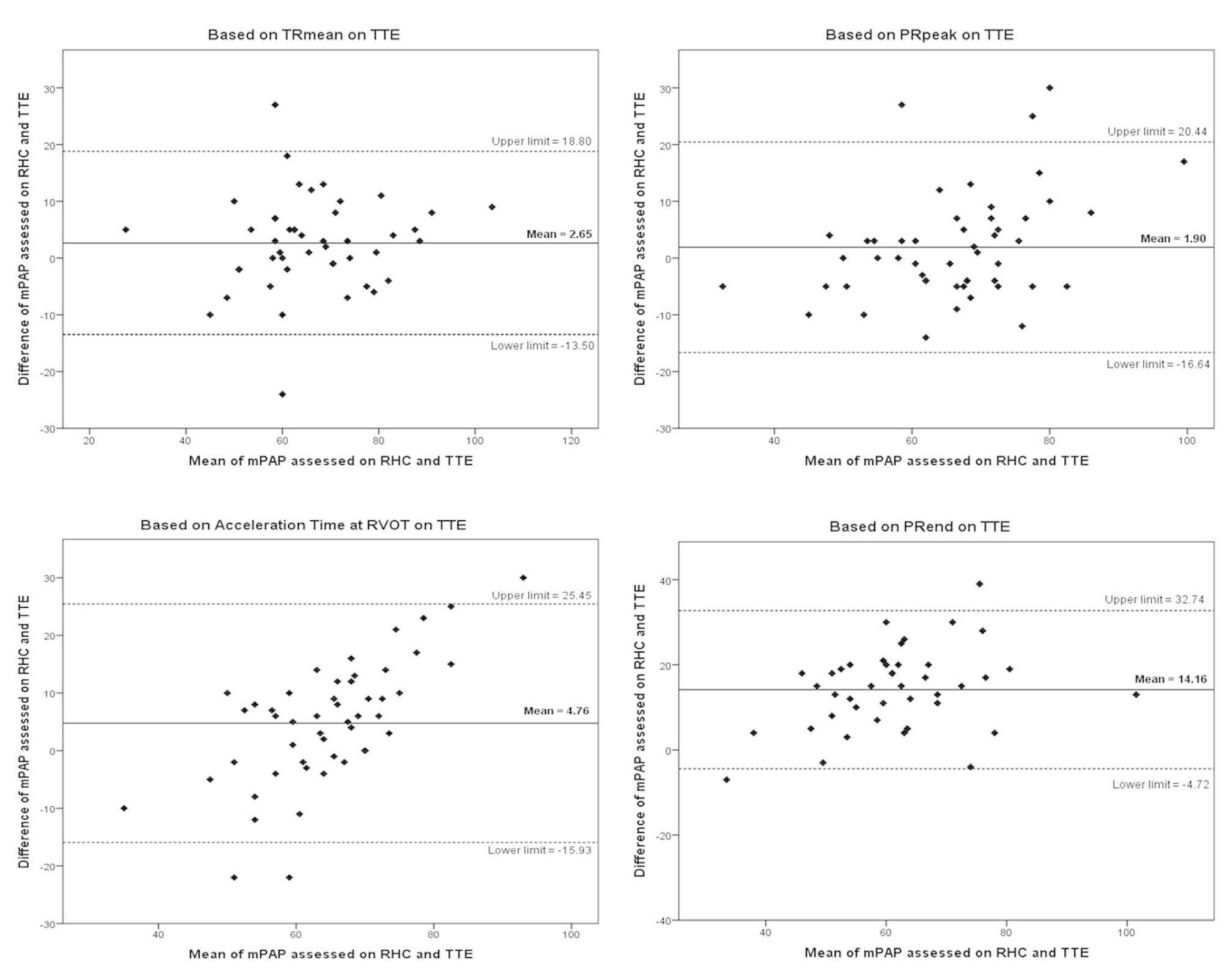
Bland-Altman plot for mean pulmonary arterial pressure (mPAP) assessed on cardiac catheterization and transthoracic echocardiogram (TTE) TTE=transthoracic echocardiography, RHC=right heart catheterization, mPAP=mean pulmonary artery pressure, TRmean=mean tricuspid regurgitation, PRpeak=peak pulmonary regurgitation, RVOT=right ventricular outflow tract, PRend=end pulmonary regurgitation

Similarly, Bland-Altman plots for mPAP assessed on cardiac catheterization and four parameters of echocardiography showed a strong correlation. Among the four methods for the assessment of mPAP on echocardiography, PGTRmean method showed relatively better agreement with a mean difference of 2.65 mmHg and 95% agreement limits of -13.5 to 18.8 mmHg, as shown in Figure 2.

## Discussion

Accurate assessment of pulmonary hypertension (PH) without any invasive modality is quite challenging in clinical practice [7]. Transthoracic echocardiography is the potential non-invasive modality for the assessment of pulmonary artery pressures in patients with PH [13]. However, a systematic review and meta-analysis of 29 studies reported a modest correlation and accuracy of echocardiography as compared to cardiac catheterization for assessment of sPAP [7]. Studies further suggest echocardiography can be used for initial assessment and screening of the patients.

Similarly, Greiner et al. found echocardiographic assessment of sPAP closely related to that assessed by the invasive method. Non-invasive diagnosis of pulmonary hypertension with Doppler echocardiography had a good sensitivity (87%), specificity (79%), and accuracy (85%) for sPAP at cut-off value of 36 mmHg, and area under the curve (AUC) of receiver operating characteristic (ROC) curve was 0.91 (P<0.001, CI 0.90 to 0.93). It was further reported that Pearson’s correlation coefficient between echocardiography and cardiac catheterization for the assessment of sPAP was 0.87 (P<0.001) [8].

Fisher et al. conducted a study to assess the utility of the echocardiography as a non-invasive modality for the diagnosis of PH. Despite optimization of the assessment methodology for potential bias, they found significant differences in estimates of pulmonary artery pressures by echocardiography and cardiac catheterization [14]. The Bland-Altman analysis showed overestimation as well as an underestimation tendency of echocardiography for the estimation of sPAP [8].

Results for our population are not different from other studies. The correlation coefficient was found to be 0.917 (p<0.001) between sPAP assessed on cardiac catheterization and echocardiography. Slightly higher correlation in our study as compared to Greiner et al. can be attributed to the fact that we excluded population with normal PAP due to the ethical concern of invasive procedure (cardiac catheterization) without proper indication [8]. However, Bland-Altman plot for sPAP assessed on two modalities (Figure 1) showed similar patterns as Fisher et al. with mean difference or bias of 1.74 mmHg and 95% limits of agreement of -13.42 to +16.90 mmHg [14].

Similarly, the correlations between cardiac catheterization and different parameters of echocardiography for the assessment of mPAP were found to be 0.832 (p<0.001), 0.749 (p<0.001), 0.691 (p<0.001), and 0.752 (p<0.001) for PGTRmean, PGPRpeak, Acceleration Time at RVOT, and PGPRend respectively. Bland-Altman plots for mPAP (Figure 2) showed relatively good agreement for TRmean method with a mean difference of 2.65 mmHg and 95% agreement limits of -13.5 to 18.8 mmHg. Our study showed that transthoracic echocardiography can be used to assess pulmonary artery pressures with reasonable accuracy. A study conducted by Er et al. reported high accuracy of mean pulmonary artery pressure (mPAP) for the assessment of PH, a cut off value of 25.5 mmHg was found to be 98% accurate, 98% sensitive, and 100% specific [15].

As the European society of cardiology has defined pulmonary hypertension on the basis of mean pulmonary artery pressure in patients with congenital heart diseases (shunt lesion) with pulmonary hypertension, operability depends on pulmonary vascular resistance which is calculated from mean pulmonary artery pressure on cardiac catheterization. Therefore, mean pulmonary artery pressure should be assessed along with systolic pulmonary artery pressure in pulmonary hypertension patient [1].

As PGTRmean and Acceleration Time across RVOT are not in routine use for pulmonary artery pressure assessment, and as the present study shows that tricuspid and /or pulmonary regurgitation was not present in all patients, there is need for a parameter that is not dependant on these factors. As Acceleration time across RVOT showed good correlation with cardiac catheterization for assessment of mPAP, it can be used in situations in which no TR and PR jet are present. Although PGTRmean is not in routine use, our study showed relatively good agreement between mPAP assessed by cardiac catheterization and TTE; therefore, it should be used routinely.

## Conclusions

A positive but modest correlation was observed between hemodynamic parameters of transthoracic echocardiography and cardiac catheterization for assessment of pulmonary artery pressures. Transthoracic echocardiography can be reliably used as an initial non-invasive modality for the assessment of pulmonary artery hypertension, and can obviate the need of right heart catheterization in some patients, especially those with mild pulmonary hypertension.
